# Representation Learning and Spectral Clustering for the Development and External Validation of Dynamic Sepsis Phenotypes: Observational Cohort Study

**DOI:** 10.2196/45614

**Published:** 2023-06-23

**Authors:** Aaron Boussina, Gabriel Wardi, Supreeth Prajwal Shashikumar, Atul Malhotra, Kai Zheng, Shamim Nemati

**Affiliations:** 1 Division of Biomedical Informatics University of California, San Diego La Jolla, CA United States; 2 Department of Emergency Medicine University of California San Diego San Diego, CA United States; 3 Division of Pulmonary, Critical Care and Sleep Medicine University of California San Diego San Diego, CA United States; 4 Department of Informatics University of California, Irvine Irvine, CA United States

**Keywords:** sepsis, phenotype, emergency service, hospital, disease progression, artificial intelligence, machine learning, emergency, infection, clinical phenotype, clinical phenotyping, transition model, transition modeling

## Abstract

**Background:**

Recent attempts at clinical phenotyping for sepsis have shown promise in identifying groups of patients with distinct treatment responses. Nonetheless, the replicability and actionability of these phenotypes remain an issue because the patient trajectory is a function of both the patient’s physiological state and the interventions they receive.

**Objective:**

We aimed to develop a novel approach for deriving clinical phenotypes using unsupervised learning and transition modeling.

**Methods:**

Forty commonly used clinical variables from the electronic health record were used as inputs to a feed-forward neural network trained to predict the onset of sepsis. Using spectral clustering on the representations from this network, we derived and validated consistent phenotypes across a diverse cohort of patients with sepsis. We modeled phenotype dynamics as a Markov decision process with transitions as a function of the patient’s current state and the interventions they received.

**Results:**

Four consistent and distinct phenotypes were derived from over 11,500 adult patients who were admitted from the University of California, San Diego emergency department (ED) with sepsis between January 1, 2016, and January 31, 2020. Over 2000 adult patients admitted from the University of California, Irvine ED with sepsis between November 4, 2017, and August 4, 2022, were involved in the external validation. We demonstrate that sepsis phenotypes are not static and evolve in response to physiological factors and based on interventions. We show that roughly 45% of patients change phenotype membership within the first 6 hours of ED arrival. We observed consistent trends in patient dynamics as a function of interventions including early administration of antibiotics.

**Conclusions:**

We derived and describe 4 sepsis phenotypes present within 6 hours of triage in the ED. We observe that the administration of a 30 mL/kg fluid bolus may be associated with worse outcomes in certain phenotypes, whereas prompt antimicrobial therapy is associated with improved outcomes.

## Introduction

Sepsis is a life-threatening condition caused by a dysregulated host immune response to infection and remains a substantial cause of morbidity and mortality worldwide [1-4]. Attempts to improve care and decrease mortality have focused on tools designed to provide timely and protocolized care to patients with sepsis. However, there is concern that this approach may be suboptimal for some patients and does not address the heterogeneity of the condition [5,6]. Clinical phenotypes, or subtypes, can be best defined as distinct groups of patients with similar laboratory abnormalities, organ dysfunction, and outcomes [7-9]. Efforts to identify clinical sepsis phenotypes have gained interest recently but have remained limited in clinical applications. The identification of clinical phenotypes early in sepsis may allow for more precision care as providers may tailor therapies (ie, quantity of fluid resuscitation or timing of vasoactive medications) based on a particular phenotype. Seymour et al [1] described 4 novel distinct clinical phenotypes of sepsis that correlated with host response patterns and clinical outcomes. However, phenotyping using unsupervised clustering on high-dimensional features is sensitive to noise, which complicates the generalizability of the associated phenotypes [10-12]. The common sources of variations and noise include differences in local populations, electronic medical record systems, laboratory equipment and assays, frequency of data measurement, and variations in clinical workflows and administrative practices, which collectively result in data distribution shifts. Rather than relying on arbitrary criteria (ie, quick Sequential Organ Failure Assessment [SOFA] or Systemic Inflammatory Response Syndrome criteria), representation learning provides a principled strategy for deriving informative compressed summaries or encodings from underlying clinical data [13,14]. This approach allows for dimensionality reduction and developing features that can then be used for downstream clustering and phenotype discovery.

Sepsis phenotypes may provide an opportunity for more personalized care and improving patient outcomes. The examples of individualized care that may be afforded by the identification of phenotypes may include varying quantities of crystalloid during resuscitation, earlier use of vasoactive medications, and earlier times for antibiotic administration. Differences in prognosis and treatment response between phenotypes may have a major clinical impact, and it would be important to identify such phenotypes soon after hospital presentation to guide treatment. Importantly, it is uncertain if these phenotypes are “static,” namely, constant throughout a patient’s stay, or “dynamic,” and sepsis interventions, such as timely antibiotic administration and fluid resuscitation, may impact phenotype membership.

We seek to classify patients meeting sepsis 3 criteria in the emergency department (ED) within the first 6 hours of arrival into distinct clinical phenotypes and compare the differential impact of sepsis therapies among phenotypes on patient-centered outcomes. We also test the hypothesis that sepsis phenotypes are dynamic, namely, that transitions between phenotypes are common over time and early sepsis interventions may impact transitions between phenotypes.

## Methods

### Study Design and Setting

We conducted a retrospective observational study consisting of all adults (aged ≥18 years) who were admitted from the ED with sepsis from the University of California, San Diego (UCSD) between January 1, 2016, and January 31, 2020, and the University of California, Irvine (UCI) between April 11, 2017, and April 8, 2022. UCSD consists of 2 EDs with a total annual census of 70,000 patients; one ED receives patients at a quaternary care center, whereas the other functions in a “safety net” hospital. UCI has a single ED with a total annual census of 50,000 patients.

### Ethics Approval

We followed recommendations provided by STROBE (Strengthening the Reporting of Observational Studies in Epidemiology) guidelines to ensure appropriate reporting of this research (Table S1 in Multimedia Appendix 1) [15]. UCSD institutional review board approval was obtained with waiver of informed consent (#800257). Data abstracted from UCI were provided under Data Use Agreement (#37533).

### Selection of Participants

All patients admitted to the hospital from the ED were automatically queried for the presence of sepsis in the electronic health record (EPIC). Data, including demographics, comorbidities, vital signs, laboratory results, medications administered (including antimicrobials and intravenous fluids), length of stay, and outcomes, were abstracted into a HIPAA (Health Insurance Portability and Accountability Act)-compliant enclave for analysis. Also included in this repository was each patient’s SOFA and Charlson Comorbidity Index. Sepsis was defined according to the Third International Consensus Definition (“sepsis 3”) as clinical suspicion of infection plus end-organ damage, represented by a 2-point change in the SOFA score [16]. Clinical suspicion of infection was defined by an emergency medicine physician ordering blood cultures and administration of at least 3 days of antibiotic therapy (excluding prophylactic use). The time of sepsis was defined as the time of clinical suspicion of infection. To accomplish this, we used the earliest timestamp of either administration of intravenous antibiotics or checking blood cultures. We included patients who met sepsis criteria within 6 hours of ED arrival and were admitted to the hospital. We excluded patients where the time of sepsis criteria was detected after evaluation in the ED; those who developed sepsis >6 hours after ED triage; those who discharged from the ED, died, or were transferred to another hospital within 6 hours of triage; and patients who had intravenous antibiotics administered for less than 3 days.

### Data Abstraction, Missingness, and Processing

Data were abstracted at the time of the first measurement in the ED. Demographic and other static data (ie, sex, weight, etc) were held constant after initial abstraction. Dynamic data points, such as blood pressure and laboratory values, were sampled and updated at hourly intervals. If a bin contained multiple values within an hour, we selected the median value. We used a sample-and-hold interpolation if data were missing. If a bin did not have any value imputed during stay in the ED, we chose the global population mean value and inserted that value in the appropriate time bin. The list of input features is provided in Table S2 in Multimedia Appendix 1. All data features first underwent normality transformations and then underwent standardization by subtracting the mean and dividing by the SD. Continuous variables are provided as medians with 25% and 75% IQRs. Binary variables are provided as percentages.

### Statistical Methods

Forty commonly used clinical variables including demographic data, vital signs, and laboratory results (see Table S2 in Multimedia Appendix 1 for a complete list of variables) were used for cluster derivation. We considered two time points per patient: (1) 3 hours after ED triage and (2) 6 hours after ED triage. We used a spectral clustering algorithm with Gaussian similarity due to its strong clustering performance on nonconvex regions [17]. In spectral clustering, data are represented by nodes in a graph with weighted edges corresponding to the similarity between nodes. Clustering is then performed on the top eigenvectors from a matrix derived from the edges. This approach recovers the largest connected components on the graph, and therefore a cluster corresponds to tightly connected subgraphs. A key advantage of spectral clustering is that it can recover clusters in nonlinear manifolds where simpler K-means clustering fails. We combined the spectral clustering method with the consensus clustering approach described by Seymour et al [1] to perform the clustering algorithm in an inner loop on random subsamples of the data to determine the consistency of the clustering results across varying values of the cluster number. We performed this analysis on both the preprocessed clinical features and the lower dimensional representations of these data from a feed-forward neural network trained to predict the onset of sepsis (Figures S1-S4 in Multimedia Appendix 1). The neural network architecture and performance are described in detail in the paper by Shashikumar et al [18].

To investigate the dynamics of the derived phenotypes, we examined the trajectory of patients from 3 to 6 hours after ED triage. We modeled changes in phenotypes as a Markov decision process and calculated transition probabilities between phenotypes (or states) as a function of the patient’s current state and a vector of actions (eg, the administration of hemodynamic resuscitation parameters and antibiotics). Specifically, we fit logistic regression models to each potential state transition using physiological and interventional features. Interventional features included the administration of antibiotics within 3 hours of ED arrival, the administration of fluids within 3 hours of ED arrival, and whether the volume of fluids administered was ≥30 mL/kg of body weight. These binary features were selected because they imitate elements of The Severe Sepsis and Septic Shock Management Bundle (SEP-1) bundle and can capture nonlinearities in the underlying continuous measurements. Statistical methods are described further in Multimedia Appendix 1.

Results were validated against an external cohort of patients with sepsis from a second clinical site (UCI). Phenotype membership was assigned using the k-nearest neighbor algorithm with the same Gaussian similarity metric as used in phenotype derivation. Phenotype characteristics and dynamics were then compared between the derivation and validation set to ensure consistency of the phenotyping. All analyses were performed using R version 4.0.4 (R Foundation for Statistical Computing).

## Results

### Characteristics of Study Subjects

We identified 11,519 patients who met criteria for sepsis within 6 hours of UCSD ED arrival between January 1, 2016, and January 31, 2020, and 2091 patients who met criteria for sepsis within 6 hours of UCI ED arrival between April 11, 2017, and April 8, 2022. Table 1 shows baseline characteristics and therapies administered to the patients included in this investigation. Most patients had some degree of chronic comorbidity (median Charlson Comorbidity Index of 2) and acute organ dysfunction (median SOFA score of 2). Almost all patients (10,094/11,519, 87.6%) received antibiotic therapy within 6 hours of identification of ED arrival. The median time to administration of antibiotics was 2.2 hours (range 1.12-4.05 hours) from ED triage. The median time to initiation of fluids was 0.87 hours, and the average quantity of fluid administered within 3 and 6 hours was 1084 mL and 1825 mL, respectively. Overall, in-hospital mortality and transition to hospice was 6.9% (800/11,519). Patients at the external validation site had similar characteristics (Table 1). Most patients received antibiotic therapy within 6 hours (median time of 2.52 hours). The median time to fluid administration was 1.29 hours. Overall, in-hospital mortality and transition to hospice was 13.4% (280/2091).

**Table 1:** Demographics, clinical characteristics, and therapies of patients with sepsis.

Variable					UCSD^a^ (development site)		UCI^b^ (validation site)
**Characteristics**							
	Patients, n			11,519			2091
	Age (years), mean (SD)			61 (17.7)			60 (17)
	**Sex, n (%)**						
		Male	6509 (56.51)			1152 (55.09)	
		Female	5010 (43.49)			939 (44.91)	
**Organ dysfunction, median (IQR)**							
	Charlson Comorbidity Index				2 (0-4)		2 (0-4)
	SOFA^c^ score (maximum within 3 hours)				2 (1-3)		2 (1-4)
**Interventions**							
	Antibiotics within 3 hours, n (%)				7250 (62.94)		1228 (58.73)
	Antibiotics within 6 hours, n (%)				10,094 (87.63)		1805 (86.32)
	Fluids, n (%)				11,512 (99.94)		1546 (73.94)
	Time to antibiotics (hours), median (IQR)				2.2 (1.12-4.05)		2.52 (1.28-4.38)
	Time to fluids (hours), median (IQR)				0.87 (0.433-1.85)		1.29 (0.45-5.66)
	Fluid intake (mL), first 3 hours, median (IQR)				1084.1 (266.56-2199.53)		500 (0-1000)
	Fluid intake ≥30 cc/kg, n (%)				3034 (26.34)		164 (7.84)
	Steroid administration, n (%)				689 (5.98)		135 (6.46)
**Laboratory values, median (IQR)**							
	Lactate (mmol/L)				2.2 (1.5-3.1)		2.2 (1.5-3.1)
**Outcomes, n (%)**							
	Mortality				715 (6.21)		108 (5.16)
	Hospice				85 (0.74)		172 (8.23)
	Septic shock				1253 (10.88)		230 (10.99)
	Acute kidney injury				3479 (30.20)		819 (39.17)
	Mechanical ventilation				993 (8.62)		181 (8.66)

^a^UCSD: University of California, San Diego.

^b^UCI: University of California, Irvine.

^c^SOFA: Sequential Organ Failure Assessment.

### Phenotype Characteristics

Consensus clustering outputs demonstrated more consistent clusters from spectral clustering on the lower dimensional representations than clustering on the preprocessed clinical features (Figures S1-S4 in Multimedia Appendix 1). Therefore, the representation-based clusters were used to identify sepsis phenotypes. Additionally, the consensus cumulative density function for clustering on the representations determined an optimal value of k=4 (Figure S2 in Multimedia Appendix 1). These 4 distinct sepsis phenotypes (P1, P2, P3, and P4) are shown in Table 2 and Table S3 in Multimedia Appendix 1. Cluster membership 3 hours after ED arrival ranged from 13.6% (1563/11,519; P2) to 36% (4152/11,519; P4), and significant variability in the clinical characteristics of patients and rates of adverse outcomes was observed across the 4 phenotypes. P3 exhibited the worst outcomes in terms of in-hospital mortality (179/1912, 9.4%), progression to septic shock (458/1912, 24%), development of acute kidney injury (AKI; 708/1912, 37%), and need for mechanical ventilation (225/1912, 11.8%). Conversely, P2 exhibited the lowest rate of in-hospital mortality (26/1563, 1.7%), septic shock (37/1563, 2.4%), AKI (393/1563, 25.1%), and mechanical ventilation (65/1563, 4.2%). Chord diagrams of the impacted body systems across these phenotypes show that the derived phenotypes express complex differences across nearly all systems (Figure S5 in Multimedia Appendix 1). Further, cluster membership is not solely a function of missingness (Table S4 in Multimedia Appendix 1). The phenotype characteristics of the k-nearest neighbor assigned clusters for patients in the validation cohort demonstrate similar patterns of outcomes and clinical variables (Tables S5 and S6 in Multimedia Appendix 1).

**Table 2:** Characteristics and therapeutics of the 4 sepsis phenotypes at the development site.

Variable				P1		P2	P3		P4		Total		*P* value^a^	
**Characteristics**														
	Patients, n (%)			3892 (33.79)		1563 (13.57)	1912 (16.60)		4152 (36.04)		11,519 (100)		N/A^b^	
	Age (years), mean (SD)			61 (17.5)		63 (17.7)	60 (17.9)		59 (17.7)		61 (17.7)		<.001	
	**Sex, n (%)**													<.001
		Male	2164 (55.60)		819 (52.40)		1178 (61.61)	2348 (56.55)		6509 (56.51)		N/A		
		Female	1728 (44.40)		744 (47.60)		734 (38.39)	1804 (43.45)		5010 (43.49)		N/A		
**Organ dysfunction**														
	**Charlson Comorbidity Index, median (IQR)**			2 (1-4)		2 (0-4)	3 (1-5)		2 (1-5)		2 (1-4)		<.001	
		Congestive heart failure component, n (%)	523 (13.44)		211 (13.50)		197 (10.30)	444 (10.69)		1375 (11.94)		<.001		
		Moderate or severe liver disease component, n (%)	170 (4.37)		45 (2.88)		121 (6.33)	184 (4.43)		520 (4.51)		<.001		
		Renal disease component, n (%)	653 (16.78)		213 (13.63)		405 (21.18)	688 (16.57)		1959 (17.01)		<.001		
	SOFA^c^ score (maximum within 3 hours), median (IQR)			2 (0-3)		1 (0-2)	3 (1-5)		2 (1-4)		2 (1-3)		<.001	
	SOFA score (maximum within 6 hours), median (IQR)			2 (1-3)		1 (0-2)	3 (1-5)		2 (1-4)		2 (1-4)		<.001	
**Antibiotics**														
	Antibiotics within 3 hours, mm (%)			2056 (52.83)		579 (37.04)	1652 (86.40)		2963 (71.36)		7250 (62.94)		<.001	
	Antibiotics within 6 hours, n (%)			3252 (83.56)		1217 (77.86)	1841 (96.29)		3784 (91.14)		10,094 (87.63)		<.001	
	Time to antibiotics (hours), median (IQR)			2.82 (1.483-4.85)		3.73 (2.25-5.633)	1.22 (0.733-2.133)		1.87 (1.017-3.317)		2.2 (1.117-4.05)		<.001	
**Fluids**														
	Fluids, n (%)			3891 (99.97)		1557 (99.62)	1912 (100)		4152 (100)		11,512 (99.99)		<.001	
	Time to fluids (hours), median (IQR)			1.05 (0.517-2.25)		1.85 (0.783-3.45)	0.63 (0.35-1)		0.75 (0.383-1.417)		0.87 (0.433-1.85)		<.001	
	Fluid intake (mL), first 3 hours, median (IQR)			886.7 (98.61-1700)		233.3 (0-1000)	2153.5 (1121.88-2982.53)		1380.6 (556.81-2393.94)		1084.1 (266.56-2199.53)		<.001	
**Vasopressors** **, n (%)**														
	Pressors			253 (6.50)		37 (2.37)	458 (23.95)		505 (12.16)		1253 (10.88)		<.001	
**Corticosteroids^d^, n (%)**														
	Steroids			120 (3.08)		27 (1.73)	96 (5.02)		139 (3.35)		382 (3.32)		<.001	
**Laboratory values**														
	Subjects with lactate measurements, n (%)			2631 (67.60)		556 (35.57)	1863 (97.44)		3679 (88.61)		8729 (75.78)		<.001	
	Lactate (mmol/L), median (IQR)			1.9 (1.4-2.6)		1.7 (1.4-2.2)	2.6 (2-3.7)		2.2 (1.5-3.2)		2.2 (1.5-3.1)		<.001	
	Platelets (1000/μL), median (IQR)			221 (146-305)		212.5 (158-276.75)	194 (114-277)		215 (137-309)		213 (140-298)		<.001	
	Fibrinogen (mg/dL), median (IQR)			203 (162-341)		430 (267.5-430.5)	288.5 (234.5-412)		189.5 (125.5-437)		235 (155.25-426.25)		.5	
	INR^e^, median (IQR)			1.2 (1.1-1.5)		1.2 (1.1-1.4)	1.35 (1.2-1.7)		1.3 (1.2-1.6)		1.3 (1.1-1.6)		<.001	
**Outcomes** **, n (%)**														
	Mortality			186 (4.78)		26 (1.66)	179 (9.36)		324 (7.80)		715 (6.21)		<.001	
	Hospice			20 (0.51)		7 (0.45)	19 (0.99)		39 (0.94)		85 (0.74)		.04	
	Septic shock			253 (6.50)		37 (2.37)	458 (23.95)		505 (12.16)		1253 (10.88)		<.001	
	Acute kidney injury			1103 (28.34)		393 (25.14)	708 (37.03)		1275 (30.71)		3479 (30.20)		<.001	
	Mechanical ventilation			310 (7.97)		65 (4.16)	225 (11.77)		393 (9.47)		993 (8.62)		<.001	

^a^*P* values for continuous variables are based on Kruskal-Wallis rank-sum tests. *P* values for categorical variables are based on Pearson chi-square tests.

^b^N/A: not applicable.

^c^SOFA: Sequential Organ Failure Assessment.

^d^Only intravenous corticosteroids administered during the initial 6-hour time frame were included.

^e^INR: international normalized ratio.

### Phenotype Dynamics

**Figure 1:**
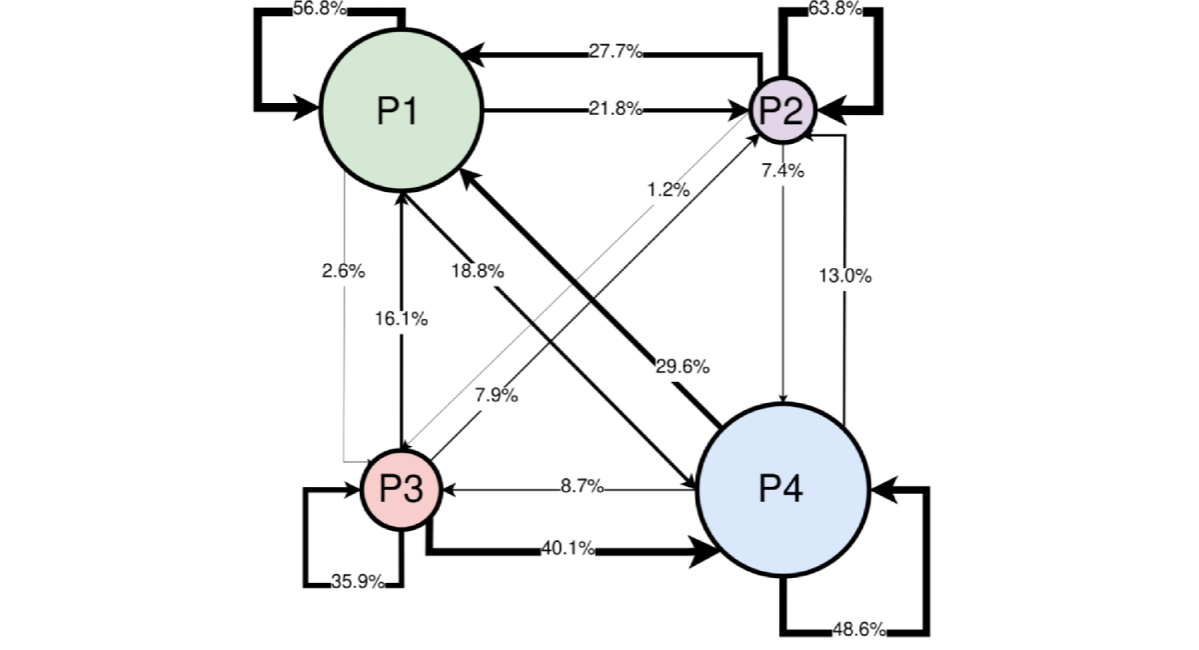
Markov phenotype transitions. The figure shows transitions between phenotypes. P1, P2, P3, and P4 represent the initial phenotype assigned at 3 hours after emergency department arrival. Transitions are noted by arrows proportional to the transition rate. Patients who remained in the same phenotype have a line that loops around to the same initial phenotype. Mortality rates per phenotype: P1 (4.8%), P2 (1.7%), P3 (9.4%), and P4 (7.8%).

The examination of patient phenotypes 3 hours after ED arrival and 6 hours after ED arrival revealed that 44.9% (5170/11,519) of patients transitioned phenotypes between these time points. Further, Markov modeling of these transitions showed that certain transitions were more favored than others (Figure 1). Transitions between phenotypes with similar rates of adverse outcomes were more likely to occur than transitions between phenotypes with larger differences. For example, the transition from the phenotype with the lowest rate of mortality, septic shock, AKI, and mechanical ventilation (P2) to the phenotype with the highest rates for these outcomes (P3) was the least likely transition, only occurring in 1.2% (11/894) of patients.

Table 3 and Tables S7-S10 in Multimedia Appendix 1 demonstrate the impact of interventions and physiological factors on predicting phenotype transitions. The results from 4 different logistic regression models (M1-M4) predicting the transition to the 4 possible states are presented. We observed that antibiotics administered within 3 hours are significantly correlated with a higher rate of transition to the lowest mortality phenotype (*P*<.001) and lower rate of transition to the second highest mortality phenotype (*P*<.001). We also observed that the fluid volume greater than 30 mL/kg of body weight is significantly correlated with a higher rate of transition to the highest mortality phenotype (*P*=.005). We did not observe these trends when evaluating transitions among SOFA groups (Tables S11 and S12 in Multimedia Appendix 1).

Table 4 and Tables S13-S16 in Multimedia Appendix 1 present the results from this analysis on the external validation data set. We observe, as in the derivation cohort, that the timely administration of antibiotics is significantly associated with transition to a low-mortality cluster (*P*=.004). We do not, however, observe any significant correlations between fluid administration greater than 30 mL/kg and phenotype transitions.

Tables S6-S12 in Multimedia Appendix 1 show the impact of interventions and physiological factors on the 16 potential phenotype transitions from one state to any other state. In the derivation and validation cohorts, we observed that patients in low-mortality phenotypes are significantly more likely to remain in that phenotype if they receive antibiotics within 3 hours of arrival. In the derivation cohort, we observe that patients in a low-mortality phenotype are more likely to transition to a high-mortality phenotype if they receive fluids within 3 hours of arrival.

**Table 3:** Interventions and physiological factors for predicting phenotype transition in the UCSD^a^ cohort.

Factor	M1^b^	M2	M3	M4
Cluster hospice+mortality, %	6.2	3.2	13.8	8.3
Average SOFA^c^ change	+0.2	+0.1	+0.6	+0.4
Antibiotics administered within 3 hours of ED^d^ triage (true/false), odds ratio (95% CI); *P* value	1.024 (0.94-1.11); .64	1.438 (1.3-1.59); <.001^e^	0.955 (0.82-1.11); .62	0.75 (0.69-0.82); <.001^e^
Fluids administered within 3 hours of ED triage (true/false), odds ratio (95% CI); *P* value	0.876 (0.78-0.98); .06	1.106 (0.97-1.27); .22	0.801 (0.62-1.03); .15	1.09 (0.95-1.25); .29
Volume of fluids within 3 hours of ED triage ≥30 mL/kg (true/false), odds ratio (95% CI); *P* value	1.067 (0.98-1.16); .21	0.938 (0.84-1.04); .32	1.227 (1.09-1.38); .005^e^	0.908 (0.84-0.99); .06
Phenotype 2, 3 hours after ED triage (true/false),^f^ odds ratio (95% CI); *P* value	0.288 (0.26-0.32); <.001	6.841 (6.11-7.65); <.001	0.444 (0.29-0.68); .001	0.329 (0.28-0.39); <.001
Phenotype 3, 3 hours after ED triage (true/false),^f^ odds ratio (95% CI); *P* value	0.144 (0.13-0.16); <.001	0.275 (0.23-0.32); <.001	21.116 (17.38-25.65); <.001	3.264 (2.93-3.64); <.001
Phenotype 4, 3 hours after ED triage (true/false),^f^ odds ratio (95% CI); *P* value	0.319 (0.29-0.35); <.001	0.501 (0.45-0.56); <.001	3.646 (3-4.43); <.001	4.374 (4.01-4.78); <.001

^a^UCSD: University of California, San Diego.

^b^M1 to M4 correspond to the 4 logistic regression models predicting the 4 possible outcomes (transition to phenotype 1, transition to phenotype 2, etc) based on the current state and interventions.

^c^SOFA: sequential organ failure assessment.

^d^ED: emergency department.

^e^*P* values <.05 represent significance.

^f^The phenotype of the patient 3 hours after ED triage.

**Table 4:** Interventions and physiological factors for predicting phenotype transition in the validation cohort.

Factor	M1^a^	M2	M3	M4
Cluster hospice+mortality, %	6.2	3.2	13.8	8.3
Average SOFA^b^ change	+0.2	+0.1	+0.6	+0.4
Antibiotics administered within 3 hours of ED^c^ triage (true/false), odds ratio (95% CI); *P* value	1.453 (1.17-1.8); .004^d^	0.932 (0.59-1.48); .80	0.99 (0.75-1.31); .95	0.757 (0.63-0.91); .02
Fluids administered within 3 hours of ED triage (true/false), odds ratio (95% CI); *P* value	1.017 (0.82-1.26); .90	1.027 (0.64-1.65); .93	1.213 (0.92-1.59); .24	0.896 (0.74-1.08); .34
Volume of fluids within 3 hours of ED triage ≥30 mL/kg (true/false), odds ratio (95% CI); *P* value	0.737 (0.46-1.17); .28	1.461 (0.34-6.32); .67	0.854 (0.55-1.33); .56	1.279 (0.9-1.81); .24
Phenotype 2, 3 hours after ED triage (true/false),^e^ odds ratio (95% CI); *P* value	0.162 (0.11-0.24); <.001	39.817 (25.03-63.34); <.001	0.924 (0.36-2.37); =.89	0.286 (0.18-0.45); <.001
Phenotype 3, 3 hours after ED triage (true/false),^e^ odds ratio (95% CI); *P* value	0.025 (0.01-0.04); <.001	0.07 (0.01-0.38); .01	32.448 (20.06-52.49); <.001	1.984 (1.53-2.57); <.001
Phenotype 4, 3 hours after ED triage (true/false),^e^ odds ratio (95% CI); *P* value	0.131 (0.1-0.16); <.001	0 (0-Inf); .98	4.169 (2.6-6.69); <.001	5.539 (4.49-6.84); <.001

^a^M1 to M4 correspond to the 4 logistic regression models predicting the 4 possible outcomes (transition to phenotype 1, transition to phenotype 2, etc) based on the current state and interventions.

^b^SOFA: sequential organ failure assessment.

^c^ED: emergency department.

^d^*P* values <.05 represent significance.

^e^The phenotype of the patient 3 hours after ED triage.

## Discussion

### Principal Results

The major findings of our paper are that using a large cohort of patients with sepsis, distinct, clinical phenotypes exist and that these phenotypes may be affected by recommended therapies. The phenotypes we identified were done with data readily available to emergency physicians at the time of treatment and were distinct from traditional sepsis classifications based on comorbidities, illness severity, and laboratory results. Such results may have an impact on early sepsis management, assist with triage of sepsis patients, and potentially improve patient-centered outcomes. Importantly, we show that these phenotypes are dynamic—that is, a significant number of patients change phenotypes after their initial cluster assignment. Our results indicate that the therapies initiated by physicians may impact the trajectory of these patients and that the administration of 30 mL/kg may be associated with worse outcomes in certain patient phenotypes.

A central finding of our work is that a large number of patients may experience a transition in their initially assigned phenotype based on therapies provided in the ED. Prior work on identifying sepsis phenotypes or subtypes has been limited to static representations of this disease process, where patients are given an initial classification without continued evaluation to determine if these phenotypes change with time or intervention. Our data suggest that these phenotypes are dynamic, namely, that patients transition between phenotypes. Specifically, we found that 48.3% of patients underwent a transition in sepsis phenotype after initial classification when we reassessed for phenotype membership. We identified that certain therapeutic interventions were associated with phenotype transition. Whether this represents the natural trajectory of patients with community-onset sepsis or host response to therapy is uncertain. However, such information may assist providers to triage patients as we show that patients rarely transition from low-mortality to high-mortality phenotypes (and vice versa) in the initial 6 hours from ED triage. It is plausible that the initiation of early sepsis therapies does indeed impact phenotypes as our data show that patients who receive prompt antimicrobials are more likely to stay in or transition to low-risk phenotypes. However, we lack biologic data (eg, gene expression and other host-response data) to establish that this change does not represent natural disease progression.

Whether a “one size fits all” approach as recommended by the current SEP-1 national quality measure is ideal remains controversial. Certain professional societies, including the American College of Emergency Medicine Physicians, have expressed concerns about the current SEP-1 bundle, specifically with regard to quantity of fluid administration and timing of antibiotics in patients without septic shock [5,19]. The current Surviving Sepsis Campaign recommends a 30 mL/kg fluid bolus for patients with sepsis-induced hypotension [20]. Prior work has described 4 distinct clinical phenotypes of patients with sepsis in the ED with distinct immune responses and clinical outcomes and showed that certain patients with sepsis may have worse outcomes with fluid resuscitation and physicians may opt for lower quantities of fluid resuscitation for patients with sepsis with certain comorbidities, such as heart failure [1,21]. Our results provide some confirmation of this, and it is possible that certain patients with sepsis may not benefit from the recommended 30 mL/kg fluid bolus currently recommended by Centers for Medicare and Medicaid Services or the Surviving Sepsis Campaign. Our data show that prompt antimicrobial therapy is associated with significant decreases in mortality and may prevent transition to higher risk phenotypes.

We recognize that the phenotypes from the external validation site did not demonstrate a phenotype with worse outcomes due to a 30 mL/kg fluid bolus. Although speculative, we hypothesize that the observed difference in the association of high-volume fluid resuscitation with state transition is due to differences in clinical practice between the sites as demonstrated by the fluid intervention differences in Table 1. Specifically, 26% of patients in the derivation cohort versus 7.8% of patients in the validation cohort received fluid volumes greater than 30 mL/kg. Alternatively, this may represent the heterogeneity of sepsis syndromes. Prospective randomized trials are needed to delineate potential patient harm (or benefit) from the quantity of fluid. Indeed, we noted a strong association between transition to the highest mortality group (P3) in patients who received this quantity of fluid within their first 6 hours in the ED in the development cohort. Patients in the phenotype who had worse outcomes (P3) shared a similar clinical profile as patients who were not harmed by fluid resuscitation. This finding may assist providers in determining which patients may benefit from additional testing (ie, dynamic tests such as the passive leg raise) to assess for fluid responsiveness rather than empiric recommendations. A recent small randomized trial showed that personalized, dynamic fluid responsiveness monitoring resulted in a significant decrease in fluid administration, with lower rates of renal replacement therapy and mechanical ventilation compared with a protocol-driven group [22].

### Limitations

We acknowledge that there are limitations to our work. Because of the retrospective nature of this study, findings are associations and we cannot show causality. Data were abstracted at predefined time points, and changes in these cutoffs may impact the clinical phenotypes provided. We excluded patients who met sepsis criteria >6 hours after their initial ED triage. Inclusion of these patients may have impacted phenotype characteristics and response to therapy. However, these patients comprised a small minority of subjects with sepsis in the ED, and, thus, we felt that their exclusion would not significantly impact our findings [23]. We did not have access to host biomarkers and transcriptomic data to confirm that these clinical phenotypes have biologic validity. Certain input variables had a high degree of missingness, and the use of the population mean for imputation of missing values may have introduced bias by overestimating the severity of illness for patients with missing values. This effect is partially counteracted by the use of representations from a neural network that considers whether a value was imputed [18].

### Conclusions

Using a development and external validation set of 11,519 and 2091 patients with sepsis, respectively, we derive and describe 4 sepsis clinical phenotypes present within 6 hours of triage in the ED. We show that 48% of patients with sepsis change phenotype membership within the first 6 hours of ED triage, which is associated with therapies provided in the ED. We observe that the administration of a 30 mL/kg fluid bolus is associated with worse outcomes in certain phenotypes, whereas prompt antimicrobial therapy is associated with improved outcomes. Additional prospective studies are needed to validate these findings, including use of genomic and other biologic data from sepsis patients to better quantify underlying pathophysiologic changes present.

## Appendix

Multimedia Appendix 1 Supplementary material.

## Abbreviations

AKI: acute kidney injury
ED: emergency department
HIPAA: Health Insurance Portability and Accountability Act
SEP-1: The Severe Sepsis and Septic Shock Management Bundle
SOFA: sequential organ failure assessment
STROBE: Strengthening the Reporting of Observational Studies in Epidemiology
UCI: University of California, Irvine
UCSD: University of California, San Diego

## Data Availability

Access to the de-identified UCSD and UCI cohorts can be made available by contacting the corresponding author and via approval from UCSD and UCI institutional review boards (and Health Data Oversight Committee).
