# Supplementary material for: Representation Learning and Spectral Clustering for the Development and External Validation of Dynamic Sepsis Phenotypes: Observational Cohort Study
Source: J Med Internet Res. 2023 Jun 23;25:e45614. doi: 10.2196/45614 (PMC10337434; doi:10.2196/45614)
Supplement: Multimedia Appendix 1 [file jmir_v25i1e45614_app1.pdf]

Development and External Validation of Dynamic Sepsis Phenotypes

Supplemental Digital Content

Table of Contents

Supplementary Methods	3
Figure S1. Consensus Matrices for Spectral Clustering on Representations	4
Figure S2. Cumulative Density Function for Spectral Clustering on Representations	5
Figure S3. Consensus Matrices for k-Means Clustering on EHR Data	6
Figure S4. Cumulative Density Function for k-means clustering	7
Table S1. STROBE Statement—checklist of items that should be included in reports of observational studies	9
Table S2. List of Clinical Variables	14
Table S3. Characteristics and Therapeutics of the 4 Sepsis Phenotypes at the Development Site (Hour 6)	15
Table S4. Performance (AUC) of Assigning Cluster Membership at ED Triage + 3 Hours Using Missing Value Flags vs True Clinical Values	17
Table S5. Characteristics and Therapeutics of the 4 Sepsis Phenotypes at the Validation Site	18
Table S6. Characteristics and Therapeutics of the 4 Sepsis Phenotypes at the Validation Site (Hour 6)	20
Table S7. Odds Ratios [95% confidence interval] (p-values) of Interventions and Physiological Factors for Predicting Phenotype Transition in the UCSD Cohort (Cluster 1)	22
Table S8. Odds Ratios [95% confidence interval] (p-values) of Interventions and Physiological Factors for Predicting Phenotype Transition in the UCSD Cohort (Cluster 2)	23
Table S9. Odds Ratios [95% confidence interval] (p-values) of Interventions and Physiological Factors for Predicting Phenotype Transition in the UCSD Cohort (Cluster 3)	24
Table S10. Odds Ratios [95% confidence interval] (p-values) of Interventions and Physiological Factors for Predicting Phenotype Transition in the UCSD Cohort (Cluster 4)	25
Table S11. Odds Ratios (p-values) of Interventions and Physiological Factors for Predicting SOFA Group Transitions in the UCSD Cohort.	26
Table S12. Odds Ratios (p-values) of Interventions and Physiological Factors for Predicting SOFA Group Transitions in the UCI Cohort.	27
Table S13. Odds Ratios [95% confidence interval] (p-values) of Interventions and Physiological Factors for Predicting Phenotype Transition in the UCI Validation Cohort (Cluster 1)	28
Table S14. Odds Ratios [95% confidence interval] (p-values) of Interventions and Physiological Factors for Predicting Phenotype Transition in the UCI Validation Cohort (Cluster 2)	29
Table S15. Odds Ratios [95% confidence interval] (p-values) of Interventions and Physiological Factors for Predicting Phenotype Transition in the UCI Validation Cohort (Cluster 3)	30
Table S16. Odds Ratios [95% confidence interval] (p-values) of Interventions and Physiological Factors for Predicting Phenotype Transition in the UCI Validation Cohort (Cluster 4)	31

Supplementary Methods

Study Design and Setting:

UCSD consists of 2 EDs with a total annual census of 70,000 patients; one ED receives patients at a quaternary care center whereas the other functions in a “safety net” hospital. UC Irvine has a single ED with a total annual census of 50,000 patients. We followed recommendations provided by STROBE guidelines to ensure appropriate reporting of this research. University of California San Diego Institutional review board (IRB) approval was obtained with waiver of informed consent (#800257). Data abstracted from University of California, Irvine was provided under Data Use Agreement #37533.

Statistical Methods:

In our modeling of phenotype dynamics as a markov decision process, only interventions within the first 3 hours from ED triage were considered so that the effect of early interventions on a patient’s trajectory could be examined. To ensure a clear separation between the variables related to transition and those related to the severity of illness, we excluded age, CCI, and SOFA from our transition models. However, we included the initial phenotype of the patient, which captures a set of features that includes age, comorbidities, and SOFA elements (among others), as a confounder to adjust for patient state. To evaluate the dynamics of our derived phenotypes against traditional patient categorizations, we also repeated this analysis using SOFA groups instead of phenotypes. Significant predictors of transition were determined from Wald tests on the odds ratios. Adjustment for multiple comparisons via the Bonferroni method was done by setting a conservative significance threshold of $p < 0.01$.

Figure S1. Consensus Matrices for Spectral Clustering on Representations

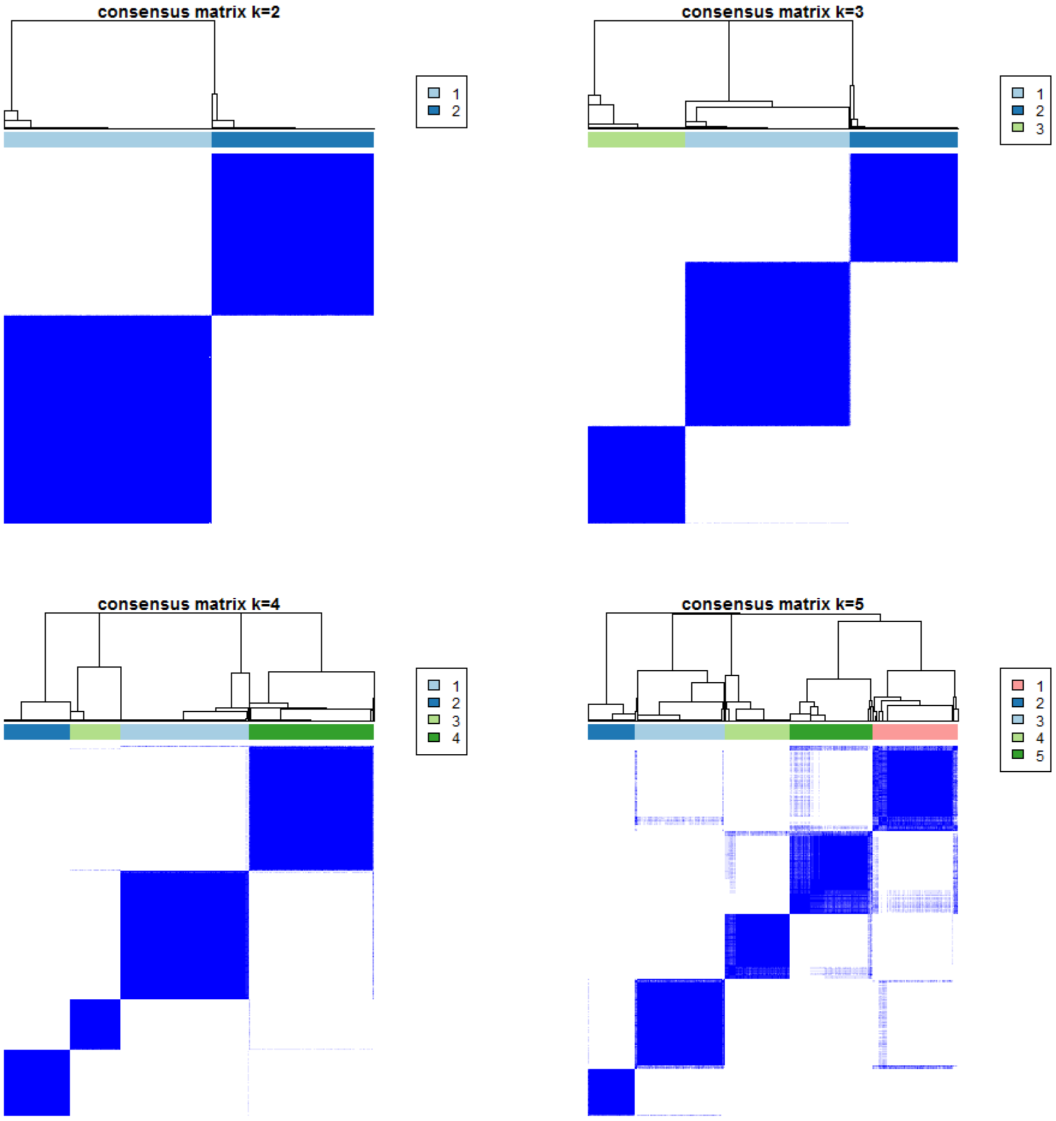

Figure S1: Consensus matrices at k=2-5 for consensus spectral clustering on the representations of a feed forward neural network trained to predict the onset of sepsis.

Figure S2. Cumulative Density Function for Spectral Clustering on Representations

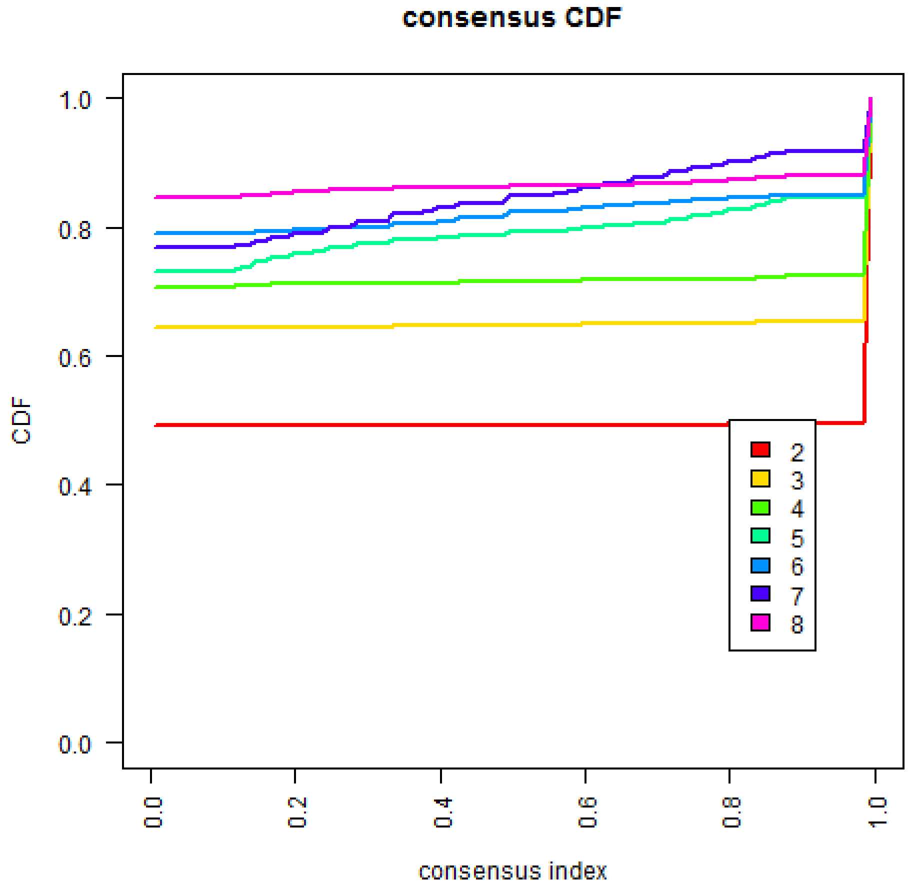

Figure S2: Cumulative density function (CDF) for consensus spectral clustering on the representations of a feed forward neural network trained to predict the onset of sepsis. To determine the optimal number of clusters (N), we utilized the CDF and increased N until the consensus index was no longer primarily 0 or 1 (i.e. the CDF curve was no longer flat). The CDF shows an optimal value of k=4.

Figure S3. Consensus Matrices for k-Means Clustering on EHR Data

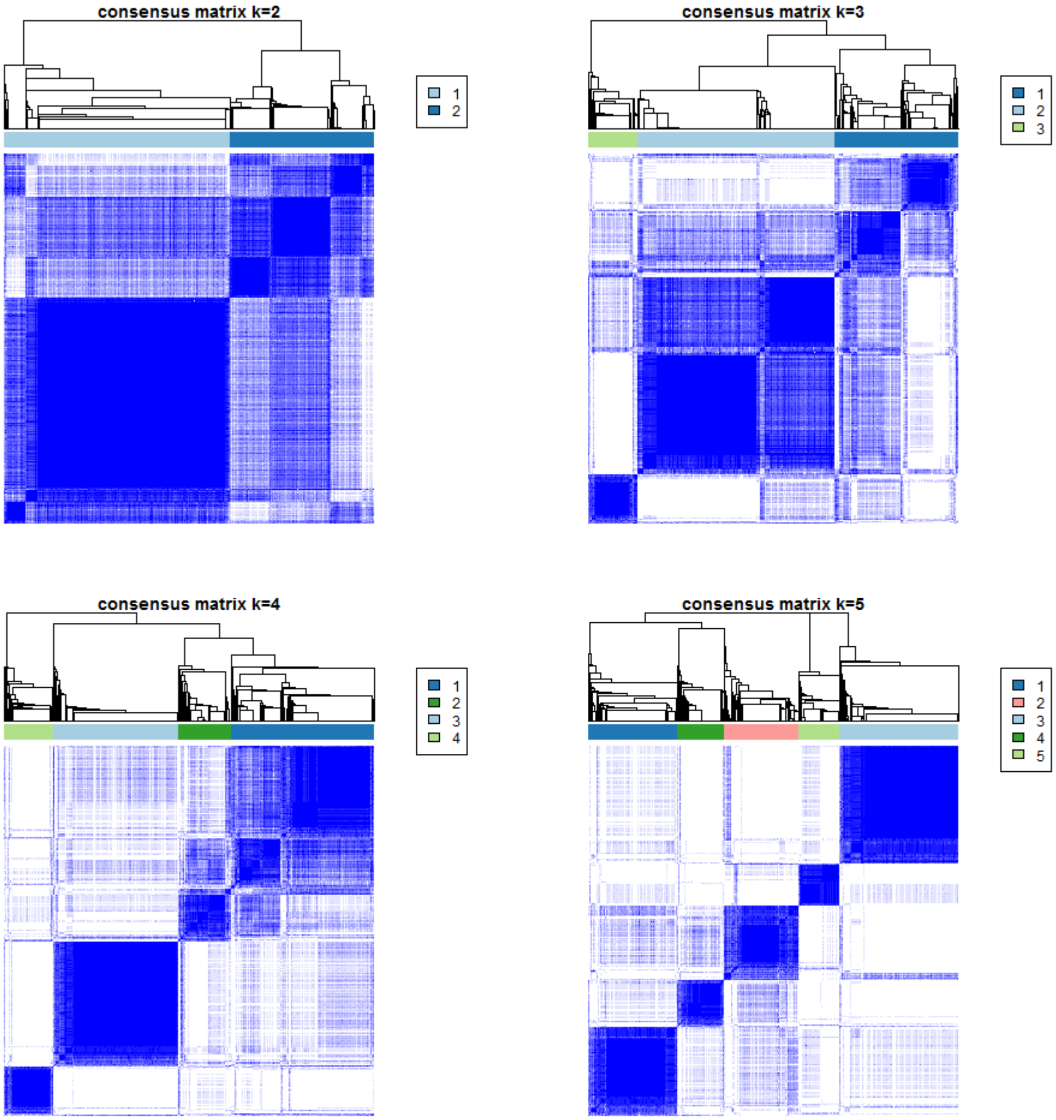

Figure S3: Consensus matrices at k=2-5 for consensus k-means clustering on normalized EHR data. The presence of cluster membership overlap between consecutive runs of k-means demonstrates that this approach results in less consistent clustering than spectral clustering on representations (Figures S1-S2).

Figure S4. Cumulative Density Function for k-means clustering

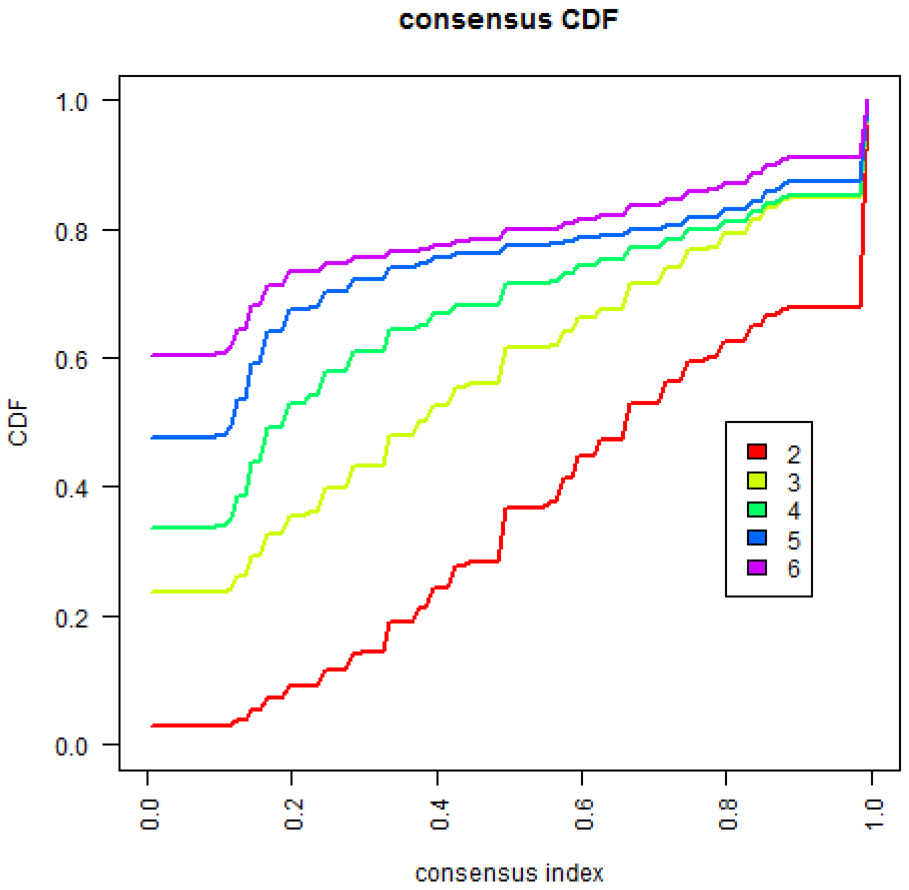

Figure S4: Cumulative density function for consensus k-means clustering on normalized EHR data.

The absence of a flattened curve demonstrates inconsistent clustering results.

Figure S5. Phenotype Chord Diagram

The chord diagram shows the clinical profile of patients in the 4 phenotypes we identified. Size of each individual ribbon represents the portion of abnormal features within that body system. For example, phenotypes 3 and 4 are more likely to have abnormal hepatic and inflammatory biomarkers compared to phenotypes 1 and 2.

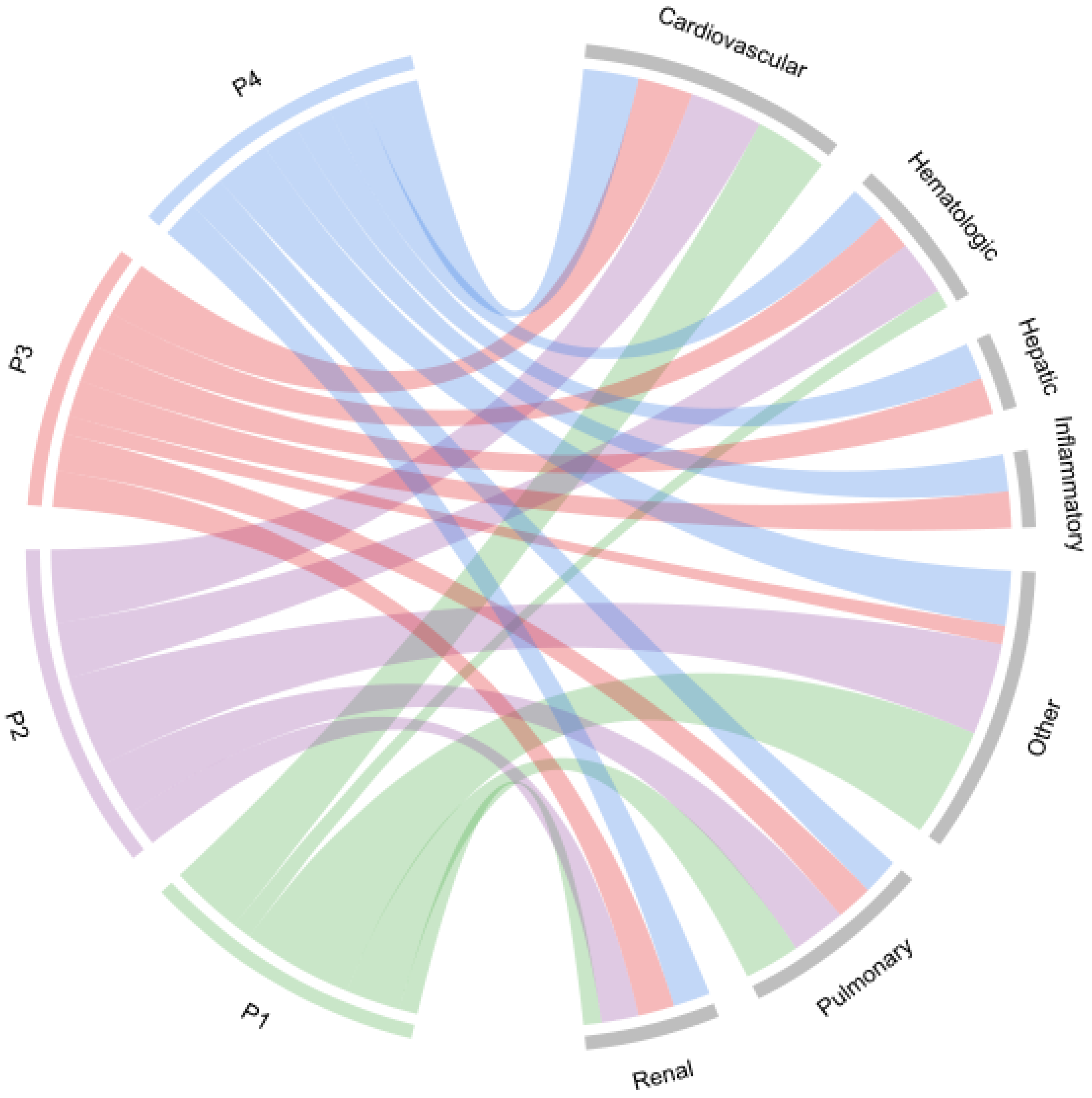

Table S1. **STROBE Statement—checklist of items that should be included in reports of observational studies**

	Item No.	Recommendation	Page No.	Relevant text from manuscript
**Title and abstract**	1	(*a*) Indicate the study's design with a commonly used term in the title or the abstract	1-3	
		(*b*) Provide in the abstract an informative and balanced summary of what was done and what was found	2-3	
**Introduction**				
Background/rationale	2	Explain the scientific background and rationale for the investigation being reported	4-5	
Objectives	3	State specific objectives, including any prespecified hypotheses	5	
**Methods**				
Study design	4	Present key elements of study design early in the paper	5-6	
Setting	5	Describe the setting, locations, and relevant dates, including periods of recruitment, exposure, follow-up, and data collection	5-6	
Participants	6	(*a*) *Cohort study*—Give the eligibility criteria, and the sources and methods of selection of participants. Describe methods of follow-up<br>*Case-control study*—Give the eligibility criteria, and the sources and methods of case ascertainment and control selection. Give the rationale for the choice of cases and controls<br>*Cross-sectional study*—Give the eligibility criteria, and the sources and methods of selection of participants	6-7	
		(*b*) *Cohort study*—For matched studies, give matching criteria and number of exposed and unexposed<br>*Case-control study*—For matched studies, give matching criteria and the number of controls per case		
Variables	7	Clearly define all outcomes, exposures, predictors, potential confounders, and effect modifiers. Give diagnostic criteria, if applicable	6 - 8	

Data sources/ measurement	8*	For each variable of interest, give sources of data and details of methods of assessment (measurement). Describe comparability of assessment methods if there is more than one group	*9*
Bias	9	Describe any efforts to address potential sources of bias	8
Study size	10	Explain how the study size was arrived at	N/a

Continued on next page

Quantitative variables	11	Explain how quantitative variables were handled in the analyses. If applicable, describe which groupings were chosen and why	8-9
Statistical methods	12	(*a*) Describe all statistical methods, including those used to control for confounding	7-9
		(*b*) Describe any methods used to examine subgroups and interactions	7-9
		(*c*) Explain how missing data were addressed	7
		(*d*) *Cohort study*—If applicable, explain how loss to follow-up was addressed<br>*Case-control study*—If applicable, explain how matching of cases and controls was addressed<br>*Cross-sectional study*—If applicable, describe analytical methods taking account of sampling strategy	N/a
		(*e*) Describe any sensitivity analyses	N/a
**Results**			
Participants	13*	(a) Report numbers of individuals at each stage of study—eg numbers potentially eligible, examined for eligibility, confirmed eligible, included in the study, completing follow-up, and analysed	10-11
		(b) Give reasons for non-participation at each stage	N/a
		(c) Consider use of a flow diagram	N/a
Descriptive data	14*	(a) Give characteristics of study participants (eg demographic, clinical, social) and information on exposures and potential confounders	10-11
		(b) Indicate number of participants with missing data for each variable of interest	Supplemental Appendix, Table S1
		(c) *Cohort study*—Summarise follow-up time (eg, average and total amount)	11
Outcome data	15*	*Cohort study*—Report numbers of outcome events or summary measures over time	*10-11*
		Case-control study—Report numbers in each exposure category, or summary measures of exposure	*N/a*

		Cross-sectional study—Report numbers of outcome events or summary measures	*N/a*
Main results	16	(*a*) Give unadjusted estimates and, if applicable, confounder-adjusted estimates and their precision (eg, 95% confidence interval). Make clear which confounders were adjusted for and why they were included	Table 1,2; 10-11
		(*b*) Report category boundaries when continuous variables were categorized	Table 1,2
		(*c*) If relevant, consider translating estimates of relative risk into absolute risk for a meaningful time period	N/a

Continued on next page

Other analyses	17	Report other analyses done—eg analyses of subgroups and interactions, and sensitivity analyses	9-11, Supplemental Figures S1-S4
**Discussion**			
Key results	18	Summarise key results with reference to study objectives	12-13
Limitations	19	Discuss limitations of the study, taking into account sources of potential bias or imprecision. Discuss both direction and magnitude of any potential bias	14-15
Interpretation	20	Give a cautious overall interpretation of results considering objectives, limitations, multiplicity of analyses, results from similar studies, and other relevant evidence	13-14
Generalisability	21	Discuss the generalisability (external validity) of the study results	13-15
**Other information**			
Funding	22	Give the source of funding and the role of the funders for the present study and, if applicable, for the original study on which the present article is based	1

*Give information separately for cases and controls in case-control studies and, if applicable, for exposed and unexposed groups in cohort and cross-sectional studies.

Note: An Explanation and Elaboration article discusses each checklist item and gives methodological background and published examples of transparent reporting. The STROBE checklist is best used in conjunction with this article (freely available on the Web sites of PLoS Medicine at http://www.plosmedicine.org/, Annals of Internal Medicine at http://www.annals.org/, and Epidemiology at http://www.epidem.com/). Information on the STROBE Initiative is available at www.strobe-statement.org

Table S2. List of Clinical Variables

Clinical Variable
**Cardiovascular**
Heart Rate
Systolic Blood Pressure
Mean Arterial Pressure
Diastolic Blood Pressure
Bicarbonate
Lactic acid
Troponin I
**Hematologic**
Hematocrit
Hemoglobin
Partial Thromboplastin Time
Fibrinogen
Platelets
**Hepatic**
Aspartate Transaminase
Alkaline Phosphatase
Bilirubin Direct
Total Bilirubin
**Inflammatory**
Temperature
Leukocyte Count
**Pulmonary**
Pulse Oximetry
Respiration rate
End tidal carbon dioxide
Fraction of inspired oxygen
Partial pressure of carbon dioxide from arterial blood
Oxygen saturation from arterial blood
**Renal**
Measure of Excess Bicarbonate
Creatinine
Blood urea nitrogen
pH
**Other**
Potassium
Calcium
Chloride
Glucose
Magnesium
Phosphate
Age
Gender

Table S3. Characteristics and Therapeutics of the 4 Sepsis Phenotypes at the Development Site (Hour 6)

	P1	P2	P3	P4	NA	Total
**Characteristic**						
Number of Patients, N (%)	4122 (35.8%)	2501 (21.7%)	1154 (10%)	3587 (31.1%)	155 (1.3%)	11519 (100%)
Age, Mean (SD)	61 (17.3)	61 (17.8)	60 (17.8)	60 (17.8)	56 (20.4)	61 (17.7)
Sex, N (%)						
Male	2294 (55.7%)	1389 (55.5%)	688 (59.6%)	2063 (57.5%)	75 (48.4%)	6509 (56.5%)
Female	1828 (44.3%)	1112 (44.5%)	466 (40.4%)	1524 (42.5%)	80 (51.6%)	5010 (43.5%)
**Organ Dysfunction**						
Charlson Comorbidity Index, Median (IQR)	2 (1-4)	2 (1-4)	2 (1-5)	2 (1-5)	2 (1-4)	2 (1-4)
CCI, Congestive Heart Failure Component, N (%)	530 (12.9%)	313 (12.5%)	116 (10.1%)	402 (11.2%)	14 (9%)	1375 (11.9%)
CCI, Moderate/Severe Liver Disease Component, N (%)	149 (3.6%)	87 (3.5%)	91 (7.9%)	190 (5.3%)	3 (1.9%)	520 (4.5%)
CCI, Renal Disease Component, N (%)	712 (17.3%)	368 (14.7%)	242 (21%)	614 (17.1%)	23 (14.8%)	1959 (17%)
SOFA Score (Max Within 3 Hours), Median (IQR)	2 (1-3)	1 (0-3)	3 (1-5)	2 (1-4)	1 (0-3)	2 (1-3)
SOFA Score (Max Within 6 Hours), Median (IQR)	2 (1-3)	2 (1-3)	3 (2-5)	2 (1-4)	1 (0-3)	2 (1-4)
**Antibiotics**						
Antibiotics Within 3 Hours, N (%)	2426 (58.9%)	1435 (57.4%)	893 (77.4%)	2370 (66.1%)	126 (81.3%)	7250 (62.9%)
Antibiotics Within 6 Hours, N (%)	3548 (86.1%)	2070 (82.8%)	1094 (94.8%)	3230 (90%)	152 (98.1%)	10094 (87.6%)

	P1	P2	P3	P4	NA	Total
Time to Antibiotics (Hrs), Median (IQR)	2.4 (1.233 - 4.383)	2.52 (1.25 - 4.65)	1.52 (0.833 - 2.783)	2.08 (1.067 - 3.767)	1.57 (0.95 - 2.592)	2.2 (1.117 - 4.05)
**Fluids**						
Fluids, N (%)	4120 (100%)	2498 (99.9%)	1154 (100%)	3587 (100%)	153 (98.7%)	11512 (99.9%)
Time to Fluids (Hrs), Median (IQR)	0.93 (0.467 - 2.004)	0.97 (0.367 - 2.175)	0.7 (0.4 - 1.246)	0.83 (0.45 - 1.617)	0.65 (-0.033 - 1.267)	0.87 (0.433 - 1.85)
Fluid Intake (mL), First Three Hours, Median (IQR)	1000 (177.01 - 2031.09)	858.2 (60 - 1776.84)	1956.7 (871.63 - 2859.19)	1270.6 (476.71 - 2321.93)	1050 (300 - 2034.17)	1084.1 (266.56 - 2199.53)
**Vasopressors**						
Pressors, N (%)	358 (8.7%)	109 (4.4%)	317 (27.5%)	459 (12.8%)	10 (6.5%)	1253 (10.9%)
**Laboratory Values**						
Subjects with Lactate Measurements, N (%)	3041 (73.8%)	1610 (64.4%)	1013 (87.8%)	2942 (82%)	123 (79.4%)	8729 (75.8%)
Lactate (mmol/L), Median (IQR)	2 (1.4 - 2.8)	1.9 (1.4 - 2.5)	2.8 (2 - 4.1)	2.3 (1.6 - 3.3)	2 (1.35 - 2.75)	2.2 (1.5 - 3.1)
**Outcomes**						
Mortality, N (%)	229 (5.6%)	67 (2.7%)	140 (12.1%)	269 (7.5%)	10 (6.5%)	715 (6.2%)
Hospice, N (%)	25 (0.6%)	14 (0.6%)	19 (1.6%)	27 (0.8%)	0 (0%)	85 (0.7%)
Septic Shock, N (%)	358 (8.7%)	109 (4.4%)	317 (27.5%)	459 (12.8%)	10 (6.5%)	1253 (10.9%)
Acute Kidney Injury, N (%)	1202 (29.2%)	665 (26.6%)	447 (38.7%)	1137 (31.7%)	28 (18.1%)	3479 (30.2%)
Mechanical Ventilation, N (%)	362 (8.8%)	122 (4.9%)	148 (12.8%)	352 (9.8%)	9 (5.8%)	993 (8.6%)

Table S4. Performance (AUC) of Assigning Cluster Membership at ED Triage + 3 Hours Using Missing Value Flags vs True Clinical Values

	Missing Value Flags	Clinical Values
**M1**	0.60	0.73
**M2**	0.80	0.91
**M3**	0.73	0.93
**M4**	0.62	0.68

Here we explore the degree to which missingness alone accounts for cluster membership. First, we train four logistic regression models to assign cluster membership at ED Triage + 3 hours based only on whether each measurement is missing or not. We then compare this against four separate models trained on the true clinical values. We report the AUCs of each of these models in the table above. As shown, severity of illness and the presence of data are indeed entangled. This is particularly apparent for M2 (the cluster 2 assignment model) which represents the healthiest phenotype. However, using information from the clinical variables significantly improves the performance of the assignment. Therefore, missingness does not fully explain cluster membership.

Table S5. **Characteristics and Therapeutics of the 4 Sepsis Phenotypes at the Validation Site**

	P1	P2	P3	P4	Total	P Value[a]
**Characteristic**						
Number of Patients, N (%)	619 (29.6%)	168 (8%)	382 (18.3%)	922 (44.1%)	2091 (100%)	-
Age, Mean (SD)	61 (17.7)	61 (18.6)	62 (17.2)	60 (17.6)	61 (17.6)	0.51
Sex, N (%)						0.39
Male	370 (59.8%)	91 (54.2%)	214 (56%)	537 (58.2%)	1212 (58%)	
Female	249 (40.2%)	77 (45.8%)	168 (44%)	385 (41.8%)	879 (42%)	
**Organ Dysfunction**						
Charlson Comorbidity Index, Median (IQR)	2 (1-4)	2 (1-4)	3 (2-5)	3 (2-5)	2 (2-5)	<0.001
CCI, Congestive Heart Failure Component, N (%)	87 (14.1%)	17 (10.1%)	35 (9.2%)	86 (9.3%)	225 (10.8%)	0.018
CCI, Moderate/Severe Liver Disease Component, N (%)	19 (3.1%)	1 (0.6%)	13 (3.4%)	45 (4.9%)	78 (3.7%)	0.03
CCI, Renal Disease Component, N (%)	106 (17.1%)	37 (22%)	82 (21.5%)	172 (18.7%)	397 (19%)	0.26
SOFA Score (Max Within 3 Hours), Median (IQR)	2 (1-3)	1 (0-2)	3 (2-5)	3 (1-4)	2 (1-4)	<0.001
SOFA Score (Max Within 6 Hours), Median (IQR)	2 (1-4)	2 (1-2)	4 (2-5)	3 (1-4)	3 (1-4)	<0.001
**Antibiotics**						
Antibiotics Within 3 Hours, N (%)	307 (49.6%)	55 (32.7%)	302 (79.1%)	564 (61.2%)	1228 (58.7%)	<0.001
Antibiotics Within 6 Hours, N (%)	517 (83.5%)	124 (73.8%)	360 (94.2%)	804 (87.2%)	1805 (86.3%)	<0.001
Time to Antibiotics (Hrs), Median (IQR)	3.02 (1.617 - 5.008)	4.15 (2.333 - 6.217)	1.62 (0.883 - 2.829)	2.35 (1.283 - 4.138)	2.52 (1.283 - 4.383)	<0.001
**Fluids**						
Fluids, N (%)	441 (71.2%)	99 (58.9%)	308 (80.6%)	698 (75.7%)	1546 (73.9%)	<0.001
Time to Fluids (Hrs), Median (IQR)	1.58 (0.583 - 5.567)	2.7 (0.625 - 6.192)	0.93 (0.417 - 3.987)	1.2 (0.433 - 5.975)	1.29 (0.45 - 5.658)	0.04
Fluid Intake (mL), First Three Hours, Median (IQR)	500 (0 - 1000)	0 (0 - 1000)	1000 (0 - 1610)	500 (0 - 1020)	500 (0 - 1000)	<0.001

	P1	P2	P3	P4	Total	P Value[a]
**Vasopressors**						
Pressors, N (%)	32 (5.2%)	4 (2.4%)	89 (23.3%)	105 (11.4%)	230 (11%)	<0.001
**Laboratory Values**						
Subjects with Lactate Measurements, N (%)	435 (70.3%)	132 (78.6%)	276 (72.3%)	629 (68.2%)	1472 (70.4%)	0.04
Lactate (mmol/L), Median (IQR)	2.1 (1.5 - 3.2)	2.1 (1.4 - 3.425)	2.2 (1.5 - 3.1)	2.2 (1.5 - 3.1)	2.2 (1.5 - 3.1)	0.98
**Outcomes**						
Mortality, N (%)	18 (2.9%)	2 (1.2%)	29 (7.6%)	59 (6.4%)	108 (5.2%)	<0.001
Hospice, N (%)	40 (6.5%)	8 (4.8%)	45 (11.8%)	79 (8.6%)	172 (8.2%)	0.008
Septic Shock, N (%)	32 (5.2%)	4 (2.4%)	89 (23.3%)	105 (11.4%)	230 (11%)	<0.001
Acute Kidney Injury, N (%)	241 (38.9%)	63 (37.5%)	159 (41.6%)	356 (38.6%)	819 (39.2%)	0.008
Mechanical Ventilation, N (%)	41 (6.6%)	11 (6.5%)	43 (11.3%)	86 (9.3%)	181 (8.7%)	<0.001

[a]P values for continuous variables are based on Kruskal-Wallis rank sum tests. P values for categorical variables are based on Pearson's χ^2 tests.

Table S6. **Characteristics and Therapeutics of the 4 Sepsis Phenotypes at the Validation Site (Hour 6)**

	P1	P2	P3	P4	NA	Total
**Characteristic**						
Number of Patients, N (%)	477 (22.8%)	121 (5.8%)	247 (11.8%)	830 (39.7%)	416 (19.9%)	2091 (100%)
Age, Mean (SD)	61 (17.2)	58 (19)	60 (16.5)	61 (17.7)	63 (17.9)	61 (17.6)
Sex, N (%)						
Male	286 (60%)	71 (58.7%)	138 (55.9%)	483 (58.2%)	234 (56.2%)	1212 (58%)
Female	191 (40%)	50 (41.3%)	109 (44.1%)	347 (41.8%)	182 (43.8%)	879 (42%)
**Organ Dysfunction**						
Charlson Comorbidity Index, Median (IQR)	2 (1-4)	2 (1-3)	3 (2-6)	2 (2-5)	2 (1-5)	2 (2-5)
CCI, Congestive Heart Failure Component, N (%)	67 (14%)	13 (10.7%)	20 (8.1%)	81 (9.8%)	44 (10.6%)	225 (10.8%)
CCI, Moderate/Severe Liver Disease Component, N (%)	15 (3.1%)	0 (0%)	11 (4.5%)	35 (4.2%)	17 (4.1%)	78 (3.7%)
CCI, Renal Disease Component, N (%)	90 (18.9%)	25 (20.7%)	58 (23.5%)	157 (18.9%)	67 (16.1%)	397 (19%)
SOFA Score (Max Within 3 Hours), Median (IQR)	2 (1-3)	1 (1-2)	3 (1-5)	2 (1-4)	3 (1-5)	2 (1-4)
SOFA Score (Max Within 6 Hours), Median (IQR)	2 (1-4)	2 (1-2)	4 (2-6)	3 (2-4)	2 (1-4)	3 (1-4)
**Antibiotics**						
Antibiotics Within 3 Hours, N (%)	272 (57%)	45 (37.2%)	174 (70.4%)	481 (58%)	256 (61.5%)	1228 (58.7%)
Antibiotics Within 6 Hours, N (%)	404 (84.7%)	92 (76%)	231 (93.5%)	716 (86.3%)	362 (87%)	1805 (86.3%)
Time to Antibiotics (Hrs), Median (IQR)	2.68 (1.35 - 4.65)	3.9 (2.15 - 5.717)	2.12 (1 - 3.225)	2.56 (1.317 - 4.446)	2.13 (1.2 - 4.15)	2.52 (1.283 - 4.383)
**Fluids**						
Fluids, N (%)	335 (70.2%)	68 (56.2%)	192 (77.7%)	643 (77.5%)	308 (74%)	1546 (73.9%)
Time to Fluids (Hrs), Median (IQR)	1.47 (0.533 - 5.6)	1.73 (0.533 - 6.458)	1 (0.446 - 4.838)	1.45 (0.517 - 6.717)	1.01 (0.262 - 3.688)	1.29 (0.45 - 5.658)
Fluid Intake (mL), First Three Hours, Median (IQR)	500 (0 - 1000)	0 (0 - 1000)	1000 (0 - 1710)	500 (0 - 1000)	500 (0 - 1000)	500 (0 - 1000)
**Vasopressors**						
Pressors, N (%)	19 (4%)	2 (1.7%)	71 (28.7%)	77 (9.3%)	61 (14.7%)	230 (11%)
**Laboratory Values**						
Subjects with Lactate Measurments, N (%)	343 (71.9%)	92 (76%)	167 (67.6%)	581 (70%)	289 (69.5%)	1472 (70.4%)

	P1	P2	P3	P4	NA	Total
Lactate (mmol/L), Median (IQR)	2.1 (1.5 - 3.25)	2 (1.375 - 3.075)	2.4 (1.45 - 3.35)	2.2 (1.5 - 3.1)	2.1 (1.5 - 3)	2.2 (1.5 - 3.1)
**Outcomes**						
Mortality, N (%)	15 (3.1%)	1 (0.8%)	22 (8.9%)	47 (5.7%)	23 (5.5%)	108 (5.2%)
Hospice, N (%)	27 (5.7%)	4 (3.3%)	27 (10.9%)	82 (9.9%)	32 (7.7%)	172 (8.2%)
Septic Shock, N (%)	19 (4%)	2 (1.7%)	71 (28.7%)	77 (9.3%)	61 (14.7%)	230 (11%)
Acute Kidney Injury, N (%)	188 (39.4%)	45 (37.2%)	114 (46.2%)	327 (39.4%)	145 (34.9%)	819 (39.2%)
Mechanical Ventilation, N (%)	27 (5.7%)	4 (3.3%)	20 (8.1%)	65 (7.8%)	65 (15.6%)	181 (8.7%)

Table S7. Odds Ratios [95% confidence interval] (p-values) of Interventions and Physiological Factors for Predicting Phenotype Transition in the UCSD Cohort (Cluster 1)

	Antibiotics administrered within 3 hours of admission (True/False)	Fluids administered within 3 hours of admission (True/False)	Volume of fluids within 3 hours of admission >= 30 mL/kg (True/False)
**M1**<br>**Cluster Mortality: 5.6%**<br>**Cluster Hospice: 0.6%**<br>**Cluster Hospice/Mortality: 6.2%**<br>**Average SOFA Change: +0.2**			
	1.03 [0.91 - 1.17] (0.67)	0.82 [0.69 - 0.96] (0.04)	0.98 [0.85 - 1.14] (0.82)
**M2**<br>**Cluster Mortality: 2.7%**<br>**Cluster Hospice: 0.6%**<br>**Cluster Hospice/Mortality: 3.2%**<br>**Average SOFA Change: +0.1**			
	1.35 [1.16 - 1.56] (0.0009)	1.34 [1.08 - 1.65] (0.0235)	0.82 [0.69 - 0.98] (0.0636)
**M3**<br>**Cluster Mortality: 12.1%**<br>**Cluster Hospice: 1.6%**<br>**Cluster Hospice/Mortality: 13.8%**<br>**Average SOFA Change: +0.6**			
	0.35 [0.23 - 0.52] (2e-05)	0.82 [0.53 - 1.25] (0.4361)	2.19 [1.4 - 3.41] (0.0037)
**M4**<br>**Cluster Mortality: 7.5%**<br>**Cluster Hospice: 0.8%**<br>**Cluster Hospice/Mortality: 8.3%**<br>**Average SOFA Change: +0.4**			
	0.79 [0.68 - 0.93] (0.014)	1.07 [0.87 - 1.31] (0.591)	1.15 [0.96 - 1.39] (0.207)

Table S8. Odds Ratios [95% confidence interval] (p-values) of Interventions and Physiological Factors for Predicting Phenotype Transition in the UCSD Cohort (Cluster 2)

	Antibiotics administrered within 3 hours of admission (True/False)	Fluids administered within 3 hours of admission (True/False)	Volume of fluids within 3 hours of admission >= 30 mL/kg (True/False)
**M1**<br>**Cluster Mortality: 5.6%**<br>**Cluster Hospice: 0.6%**<br>**Cluster Hospice/Mortality: 6.2%**<br>**Average SOFA Change: +0.2**			
	0.78 [0.62 - 0.98] (0.074)	0.9 [0.72 - 1.13] (0.438)	1.45 [0.99 - 2.14] (0.112)
**M2**<br>**Cluster Mortality: 2.7%**<br>**Cluster Hospice: 0.6%**<br>**Cluster Hospice/Mortality: 3.2%**<br>**Average SOFA Change: +0.1**			
	1.72 [1.39 - 2.12] (2.6e-05)	0.88 [0.71 - 1.09] (0.310)	0.58 [0.4 - 0.83] (0.013)
**M3**<br>**Cluster Mortality: 12.1%**<br>**Cluster Hospice: 1.6%**<br>**Cluster Hospice/Mortality: 13.8%**<br>**Average SOFA Change: +0.6**			
	0.55 [0.21 - 1.4] (0.29)	1.41 [0.55 - 3.65] (0.55)	2.09 [0.58 - 7.56] (0.34)
**M4**<br>**Cluster Mortality: 7.5%**<br>**Cluster Hospice: 0.8%**<br>**Cluster Hospice/Mortality: 8.3%**<br>**Average SOFA Change: +0.4**			
	0.38 [0.25 - 0.56] (6.2e-05)	1.9 [1.28 - 2.81] (0.0071)	1.69 [0.93 - 3.06] (0.1494)

Table S9. Odds Ratios [95% confidence interval] (p-values) of Interventions and Physiological Factors for Predicting Phenotype Transition in the UCSD Cohort (Cluster 3)

	Antibiotics administrered within 3 hours of admission (True/False)	Fluids administered within 3 hours of admission (True/False)	Volume of fluids within 3 hours of admission >= 30 mL/kg (True/False)
**M1**<br>**Cluster Mortality: 5.6%**<br>**Cluster Hospice: 0.6%**<br>**Cluster Hospice/Mortality: 6.2%**<br>**Average SOFA Change: +0.2**			
	1.15 [0.82 - 1.62] (0.48777)	0.82 [0.39 - 1.74] (0.66028)	1 [0.81 - 1.24] (0.98896)
**M2**<br>**Cluster Mortality: 2.7%**<br>**Cluster Hospice: 0.6%**<br>**Cluster Hospice/Mortality: 3.2%**<br>**Average SOFA Change: +0.1**			
	1.29 [0.79 - 2.09] (0.39)	1.28 [0.36 - 4.59] (0.75)	1.12 [0.84 - 1.49] (0.52)
**M3**<br>**Cluster Mortality: 12.1%**<br>**Cluster Hospice: 1.6%**<br>**Cluster Hospice/Mortality: 13.8%**<br>**Average SOFA Change: +0.6**			
	1.1 [0.85 - 1.41] (0.557)	0.6 [0.34 - 1.06] (0.142)	1.19 [1.01 - 1.4] (0.078)
**M4**<br>**Cluster Mortality: 7.5%**<br>**Cluster Hospice: 0.8%**<br>**Cluster Hospice/Mortality: 8.3%**<br>**Average SOFA Change: +0.4**			
	0.8 [0.63 - 1.01] (0.121)	1.75 [0.97 - 3.14] (0.118)	0.82 [0.7 - 0.96] (0.036)

Table S10. Odds Ratios [95% confidence interval] (p-values) of Interventions and Physiological Factors for Predicting Phenotype Transition in the UCSD Cohort (Cluster 4)

	Antibiotics administrered within 3 hours of admission (True/False)	Fluids administered within 3 hours of admission (True/False)	Volume of fluids within 3 hours of admission >= 30 mL/kg (True/False)
**M1**<br>**Cluster Mortality: 5.6%**<br>**Cluster Hospice: 0.6%**<br>**Cluster Hospice/Mortality: 6.2%**<br>**Average SOFA Change: +0.2**			
	1.09 [0.94 - 1.25] (0.33)	1.13 [0.88 - 1.47] (0.42)	1.09 [0.96 - 1.23] (0.26)
**M2**<br>**Cluster Mortality: 2.7%**<br>**Cluster Hospice: 0.6%**<br>**Cluster Hospice/Mortality: 3.2%**<br>**Average SOFA Change: +0.1**			
	1.36 [1.11 - 1.66] (0.012)	1.43 [0.95 - 2.16] (0.156)	1.09 [0.93 - 1.29] (0.378)
**M3**<br>**Cluster Mortality: 12.1%**<br>**Cluster Hospice: 1.6%**<br>**Cluster Hospice/Mortality: 13.8%**<br>**Average SOFA Change: +0.6**			
	1.22 [0.96 - 1.55] (0.17)	0.91 [0.6 - 1.39] (0.71)	1.11 [0.91 - 1.36] (0.38)
**M4**<br>**Cluster Mortality: 7.5%**<br>**Cluster Hospice: 0.8%**<br>**Cluster Hospice/Mortality: 8.3%**<br>**Average SOFA Change: +0.4**			
	0.77 [0.68 - 0.88] (0.0008)	0.83 [0.66 - 1.04] (0.1733)	0.86 [0.77 - 0.97] (0.0314)

Table S11. Odds Ratios (p-values) of Interventions and Physiological Factors for Predicting SOFA Group Transitions in the UCSD Cohort.

	**Antibiotics administered within 3 hours of ED arrival (True/False)**	**Fluids administered within 3 hours of ED arrival (True/False)**	**Volume of fluids within 3 hours of ED arrival >= 30 mL/kg (True/False)**	**SOFA [3-6), 3 Hours After Admission**	**SOFA [6-10), 3 Hours After Admission**	**SOFA [10 - 18), 3 Hours After Admission**
**M1** SOFA: [0 - 3)	1.042 (0.6242)	1.406 (0.0028)	0.929 (0.3798)	0.006 (<2e-16)	0.001 (<2e-16)	0 (0.9037)
**M2** SOFA: [3 - 6)	0.982 (0.8003)	0.737 (0.0026)	1.088 (0.2416)	58.797 (< 2e-16)	1.651 (4.8e-06)	0.098 (0.0208)
**M3** SOFA: [6 - 10)	0.942 (0.61)	1.096 (0.58)	0.955 (0.68)	15.028 (<2e-16)	589.352 (<2e-16)	81.364 (<2e-16)
**M4** SOFA: [10 - 18)	1.178 (0.5673)	1.121 (0.7866)	0.866 (0.5826)	8.214 (0.0012)	159.128 (<2e-16)	4725.546 (<2e-16)

At a significance threshold of $p < 0.01$, early administration of fluids is the only intervention significantly associated with transition. Since the administration of vasopressors is one of the SOFA score criteria, this may be a result of the observed inverse correlation between the use of fluids and the administration of pressors.

Table S12. Odds Ratios (p-values) of Interventions and Physiological Factors for Predicting SOFA Group Transitions in the UCI Cohort.

	**Antibiotics administered within 3 hours of ED arrival (True/False)**	**Fluids administered within 3 hours of ED arrival (True/False)**	**Volume of fluids within 3 hours of ED arrival >= 30 mL/kg (True/False)**	**SOFA [3-6), 3 Hours After Admission**	**SOFA [6-10), 3 Hours After Admission**	**SOFA [10 - 18), 3 Hours After Admission**
**M1** SOFA: [0 - 3)	1.185 (0.29)	0.87 (0.40)	1.434 (0.26)	0.008 (<2e-16)	0.002 (<2e-16)	0 (0.96)
**M2** SOFA: [3 - 6)	0.922 (0.56)	1.193 (0.22)	0.939 (0.82)	52.1 (<2e-16)	1.096 (0.71)	0 (0.97)
**M3** SOFA: [6 - 10)	0.953 (0.82)	0.941 (0.78)	0.716 (0.42)	8.124 (3.2e-10)	436.502 (< 2e-16)	108.058 (< 2e-16)
**M4** SOFA: [10 - 18)	0.734 (0.51)	0.614 (0.33)	0.673 (0.68)	0.723 (0.79)	29.86 (1.6e-05)	553.359 (2.5e-15)

At a significance threshold of $p < 0.01$, no intervention is significantly associated with transition.

Table S13. Odds Ratios [95% confidence interval] (p-values) of Interventions and Physiological Factors for Predicting Phenotype Transition in the UCI Validation Cohort (Cluster 1)

	Antibiotics administrered within 3 hours of admission (True/False)	Fluids administered within 3 hours of admission (True/False)	Volume of fluids within 3 hours of admission >= 30 mL/kg (True/False)
**M1**<br>**Cluster Mortality: 3.1%**<br>**Cluster Hospice: 5.7%**<br>**Cluster Hospice/Mortality: 8.8%**<br>**Average SOFA Change: +0.2**			
	1.639 [1.21 - 2.23] (0.0081)	1.075 [0.79 - 1.47] (0.7036)	1.256 [0.5 - 3.14] (0.6814)
**M2**<br>**Cluster Mortality: 0.8%**<br>**Cluster Hospice: 3.3%**<br>**Cluster Hospice/Mortality: 4.1%**<br>**Average SOFA Change: +0.2**			
	0.64 [0.31 - 1.33] (0.32)	0.606 [0.28 - 1.31] (0.29)	2.108 [0.34 - 13.11] (0.50)
**M3**<br>**Cluster Mortality: 8.9%**<br>**Cluster Hospice: 10.9%**<br>**Cluster Hospice/Mortality: 19.8%**<br>**Average SOFA Change: +0.7**			
	0.931 [0.39 - 2.22] (0.89)	0.859 [0.36 - 2.08] (0.78)	0 [0 - Inf] (0.99)
**M4**<br>**Cluster Mortality: 5.7%**<br>**Cluster Hospice: 9.9%**<br>**Cluster Hospice/Mortality: 15.5%**<br>**Average SOFA Change: +0.3**			
	0.64 [0.46 - 0.88] (0.02275)	1.039 [0.75 - 1.44] (0.84822)	0.761 [0.29 - 2.03] (0.64654)

Note: NAs indicate a lack of relevant data.

Table S14. Odds Ratios [95% confidence interval] (p-values) of Interventions and Physiological Factors for Predicting Phenotype Transition in the UCI Validation Cohort (Cluster 2)

	Antibiotics administrered within 3 hours of admission (True/False)	Fluids administered within 3 hours of admission (True/False)	Volume of fluids within 3 hours of admission >= 30 mL/kg (True/False)
**M1**<br>**Cluster Mortality: 3.1%**<br>**Cluster Hospice: 5.7%**<br>**Cluster Hospice/Mortality: 8.8%**<br>**Average SOFA Change: +0.2**			
	0.836 [0.39 - 1.8] (0.70)	0.565 [0.25 - 1.29] (0.26)	0 [0 - Inf] (0.99)
**M2**<br>**Cluster Mortality: 0.8%**<br>**Cluster Hospice: 3.3%**<br>**Cluster Hospice/Mortality: 4.1%**<br>**Average SOFA Change: +0.2**			
	1.12 [0.6 - 2.1] (0.766)	1.596 [0.83 - 3.08] (0.241)	NA
**M3**<br>**Cluster Mortality: 8.9%**<br>**Cluster Hospice: 10.9%**<br>**Cluster Hospice/Mortality: 19.8%**<br>**Average SOFA Change: +0.7**			
	0.596 [0.08 - 4.2] (0.66)	2.598 [0.48 - 14.15] (0.35)	0 [0 - Inf] (1.00)
**M4**<br>**Cluster Mortality: 5.7%**<br>**Cluster Hospice: 9.9%**<br>**Cluster Hospice/Mortality: 15.5%**<br>**Average SOFA Change: +0.3**			
	1.15 [0.47 - 2.8] (0.80)	0.621 [0.23 - 1.68] (0.43)	0 [0 - Inf] (0.99)

Note: NAs indicate a lack of relevant data.

Table S15. Odds Ratios [95% confidence interval] (p-values) of Interventions and Physiological Factors for Predicting Phenotype Transition in the UCI Validation Cohort (Cluster 3)

	Antibiotics administrered within 3 hours of admission (True/False)	Fluids administered within 3 hours of admission (True/False)	Volume of fluids within 3 hours of admission >= 30 mL/kg (True/False)
**M1**<br>**Cluster Mortality: 3.1%**<br>**Cluster Hospice: 5.7%**<br>**Cluster Hospice/Mortality: 8.8%**<br>**Average SOFA Change: +0.2**			
	NA	1.078 [0.37 - 3.14] (0.91)	1.161 [0.28 - 4.8] (0.86)
**M2**<br>**Cluster Mortality: 0.8%**<br>**Cluster Hospice: 3.3%**<br>**Cluster Hospice/Mortality: 4.1%**<br>**Average SOFA Change: +0.2**			
	NA	0 [0 - Inf] (1)	0.898 [0 - Inf] (1)
**M3**<br>**Cluster Mortality: 8.9%**<br>**Cluster Hospice: 10.9%**<br>**Cluster Hospice/Mortality: 19.8%**<br>**Average SOFA Change: +0.7**			
	1.034 [0.64 - 1.66] (0.91)	0.982 [0.65 - 1.49] (0.94)	1.015 [0.55 - 1.87] (0.97)
**M4**<br>**Cluster Mortality: 5.7%**<br>**Cluster Hospice: 9.9%**<br>**Cluster Hospice/Mortality: 15.5%**<br>**Average SOFA Change: +0.3**			
	0.771 [0.48 - 1.24] (0.37)	1.039 [0.68 - 1.58] (0.88)	0.959 [0.52 - 1.78] (0.91)

Note: NAs indicate a lack of relevant data.

Table S16. Odds Ratios [95% confidence interval] (p-values) of Interventions and Physiological Factors for Predicting Phenotype Transition in the UCI Validation Cohort (Cluster 4)

	**Antibiotics administrered within 3 hours of admission (True/False)**	**Fluids administered within 3 hours of admission (True/False)**	**Volume of fluids within 3 hours of admission >= 30 mL/kg (True/False)**
**M1**<br>**Cluster Mortality: 3.1%**<br>**Cluster Hospice: 5.7%**<br>**Cluster Hospice/Mortality: 8.8%**<br>**Average SOFA Change: +0.2**			
	1.301 [0.93 - 1.82] (0.196)	1.077 [0.77 - 1.51] (0.715)	0.498 [0.26 - 0.97] (0.085)
**M2**<br>**Cluster Mortality: 0.8%**<br>**Cluster Hospice: 3.3%**<br>**Cluster Hospice/Mortality: 4.1%**<br>**Average SOFA Change: +0.2**			
	1 [0 - Inf] (1)	1 [0 - Inf] (1)	1 [0 - Inf] (1)
**M3**<br>**Cluster Mortality: 8.9%**<br>**Cluster Hospice: 10.9%**<br>**Cluster Hospice/Mortality: 19.8%**<br>**Average SOFA Change: +0.7**			
	0.986 [0.66 - 1.47] (0.954)	1.538 [1.02 - 2.31] (0.082)	0.753 [0.38 - 1.48] (0.492)
**M4**<br>**Cluster Mortality: 5.7%**<br>**Cluster Hospice: 9.9%**<br>**Cluster Hospice/Mortality: 15.5%**<br>**Average SOFA Change: +0.3**			
	0.837 [0.63 - 1.11] (0.294)	0.769 [0.58 - 1.02] (0.127)	1.822 [1.09 - 3.03] (0.053)

Note: NAs indicate a lack of relevant data.
